# Effects of polycyclic aromatic hydrocarbons on the proliferation and differentiation of placental cells

**DOI:** 10.1186/s12958-022-00920-7

**Published:** 2022-03-08

**Authors:** Yun Sung Jo, Hyun Sun Ko, Ah Young Kim, Ha Gyeong Jo, Woo Jung Kim, Sae Kyung Choi

**Affiliations:** Department of Obstetrics and Gynecology, College of Medicine, The Catholic University of Korea, 222, Banpo-daero, Seocho-gu, Seoul, 06591 Republic of Korea

**Keywords:** Pregnancy, Placenta, Endocrine disruptors, Anthracene, Female reproduction, Polycyclic aromatic hydrocarbons

## Abstract

**Background:**

The purpose of this study was to investigate the effects of polycyclic aromatic hydrocarbons (PAHs) other than bisphenol A (BPA) and BPA substitutes on placental cells.

**Methods:**

HTR-8/SVneo cells were treated with anthracene, benzo[k]fluoranthene, benzo[a]pyrene, and 4,4-(9-fluorenylidene)diphenol, which is used as a substitute for BPA-free products. After confirming the dose response for each reagent using the prepared cells, the cells were incubated for 24, 48, and 72 h. Cell viability was confirmed using the XTT assay. Each experiment was performed with the minimum number of samples (*n* = 3) required for statistical analysis. The results were analyzed using t-tests; *p* < 0.05 was considered statistically significant.

**Results:**

After treatment with anthracene, benzo[k]fluoranthene, benzo[a]pyrene, and 4,4-(9-fluorenylidene)diphenol, the absorbance measured using the XTT assay decreased significantly with increasing concentration. The absorbance decreased significantly over time following treatment with each endocrine disruptor at the concentration confirmed by the dose–response analysis.

**Conclusions:**

This study showed that anthracene, benzo[k]fluoranthene, benzo[a]pyrene, and 4,4-(9-fluorenylidene)diphenol—a BPA substitute—affect cell viability and necrosis in the placental cell line. The study indicates the serious effects of PAHs that negatively affect pregnancy but were previously unknown. Further, this study would serve as a reference for the identification of harmful PAHs during pregnancy prognosis in women who are more susceptible to PAH exposure.

## Background

Endocrine disruptors are chemicals that can disrupt the endocrine system in both humans and animals. Female reproductive ability is an important process regulated by hormones and is susceptible to exposure to endocrine-disrupting substances. Endocrine disruptors have a benzene ring structure similar to that of steroid hormones among several female hormones. Therefore, they can easily affect reproductive hormones with a benzene ring structure [1, 2]. Female reproductive disorders caused by endocrine disruptors can lead to inadequate hormone production, infertility, anovulation, premature ovarian failure, and menstrual disorders. Various endocrine disruptors have been reported to disrupt the endocrine system in reproductive-aged women and to cause congenital disabilities [3, 4].

Polycyclic aromatic hydrocarbons (PAHs), which are endocrine disruptors, are common environmental pollutants released by incomplete combustion processes. PAHs emitted into our living environment are of interest because they have harmful side effects to humans, such as teratogenicity, mutagenicity, and carcinogenicity [5]. PAHs enter the body primarily through inhalation and ingestion. In pregnant women, PAHs that enter the body induce side effects on the mother, which are also transmitted through the placenta to the fetus, causing severe fetal developmental problems [6]. The growth and function of the placenta are regulated and coordinated to perform the placental role efficiently during pregnancy. If PAHs are transmitted from the mother to the fetus through the placenta, they negatively affect fetal growth. In addition, exposure to harmful substances such as PAHs during pregnancy can lead to disability even after the fetus grows into an adult [7].

A recent study analyzed the correlation between preterm labor and the concentration of PAHs in the placenta of women living in areas with high PAH exposure. This study showed that high PAH concentration, DNA products exposed to PAHs, and regulation of endocrine metabolic pathways affect preterm labor in pregnant women living near areas contaminated with environmental pollutants.

Given the results of these studies, exposure to PAHs during pregnancy is a critical issue in terms of teratogenicity, fetal growth, and preterm labor. However, there are various PAH types, and the studies so far have mainly focused on bisphenol A (BPA) and some phthalates. Therefore, we experimented on the PAHs that have not been studied for their effects on pregnancy. We examined cell viability and necrosis in a placental cell line treated with the selected PAHs. This study indicates the serious effects of PAHs that negatively affect pregnancy but were previously unknown. Moreover, the study is a reference for the identification of harmful PAHs during pregnancy prognosis in women who are more susceptible to PAH exposure.

## Methods

### Chemicals and reagents

Four PAHs, which have not been explored for their effects on pregnancy, were selected for experimentation on cell viability and necrosis in a placental cell line treated with the selected PAHs. In addition, 4,4–9(fluorenylidene)diphenol, a BPA substitute, which was recently used in BPA-free products, was also studied for its effect on cell viability and necrosis in a placental cell line.

PAHs used in this experiment are as follows:

1. Anthracene (Sigma-Aldrich, St Louis, MO USA, #07,671–100 mg)
2. Benzo[k]fluoranthene (Sigma-Aldrich, St Louis, MO USA, #03,323–10 mg)
3. Benzo[a]pyrene (Sigma-Aldrich, St Louis, MO USA, #51,968–50 mg)
4. 4,4-(9-Fluorenylidene)diphenol (BPA substitute) (Sigma-Aldrich, St Louis, MO USA, #399,981–25 g)

### Cell culture and treatment

The human placental HTR-8/SVneo cell line was provided by Queen’s University (Ontrio, Canada). HTR-8/SVneo cells were seeded in 100-mm culture plates at a density of 1 × 10^6^, followed by starvation for 24 h to increase the effectiveness of the substance to be treated. After the cells were grown up to 80% confluency in the plate containing RPMI 1640 medium without fetal bovine serum (Gibco, CAT NO. #21,875,034), the serum-free medium was removed, the cells were washed lightly with phosphate-buffered saline to remove all traces of the medium, and were then treated with trypsin/EDTA. After treatment with trypsin/EDTA for 1 min, the plate was tapped lightly on one side for the detachment of the cells from the bottom of the plate. After removing the cells, 10 mL of serum-free medium was evenly sprayed into the plate to remove all remaining cells from the bottom, and the solution containing the cells was collected, and transferred to a 15-mL Eppendorf tube. The solution containing the cells was centrifuged at 200 × g for 10 min (Centrifuge 5810r, Eppendorf). After centrifugation, we the supernatant was discarded and 4 mL of RPMI 1640 medium was added to the pellet. Then, 1 mL of cell suspension was transferred into a microtube, and the cells were detached once again, following which, 20 µL of the cell suspension was transferred into another microtube. After the sufficient mixing of the cell suspension, 20 µL of the cell suspension was injected into the narrow groove of the prepared hemocytometer using a pipette. We counted the cells in 4 squares of the hemocytometer mounted on a microscope at × 10 magnification. The diluted cells of 100 µL each were transferred in three compartments of 96-well microtiter plates in triplicate. Only the cell-free medium (100 µL) was separately added into the three compartments and was used as a blank. The seeded plates were incubated for 12 h at 37 °C in 5% CO_2_. After confirming that the cells were attached to the plate using a microscope, the cells were treated with each PAH separately and cultured.

### Dose response

Prior to the experiment, we attempted to determine the effective concentration of each PAH to use in time-response experiments with PAHs. The prepared cells were incubated for 24 h at predetermined concentrations (Table 1) [8–10]. After dissolving PAHs in dimethyl sulfoxide, they were diluted with RPMI 1640 medium at different concentrations. The cells were cultured with 0 µg/mL, 0.05 µg/mL, 0.5 µg/mL, 5 µg/mL, and 50 µg/mL of anthrane; 0 µg/mL, 0.3 µg/mL, 3 µg/mL, 30 µg/mL, and 300 µg/mL of benzo[k]fluoranthene; 0 µg/mL, 0.1 µg/mL, 1 µg/mL, 10 µg/mL, and 100 µg/mL of benzo[a]pyrene; 0 µg/mL, 0.005 µg/mL, 0.05 µg/mL, 0.5 µg/mL, and 5 µg/mL of 4,4-(9-Fluorenylidene)diphenol. Cell growth and viability were examined by measuring absorbance using the XTT assay (XTT Cell Proliferation Assay Kit, American Type Culture Collection, Manassas, VA, USA) at 450–650 nm. The concentration of PAH showing a statistically significant change in optical density (OD) was compared with the respective control.

**Table 1:** Absorbance of HTR8/SVneo according to the exposure dose of the endocrine disruptors

Materials	Doses (μg/mL)	O.D. (Individuals)				O.D. (Total, *n* = 4)
**Anthracene**	0	1.502	1.534	1.572	1.510	1.530 ± 0.029
	0.05	1.500	1.520	1.566	1.475	1.515 ± 0.036
	0.5	1.469	1.501	1.508	1.400	1.470 ± 0.046^*^
	5	1.414	1.469	1.453	1.345	1.420 ± 0.051^**^
	50	1.385	1.419	1.399	1.260	1.366 ± 0.067^**^
**Benzo[k]fluoranthene (B[k]F)**	0	1.543	1.555	1.570	1.552	1.555 ± 0.010
	0.3	1.569	1.574	1.564	1.560	1.567 ± 0.006
	3	1.546	1.539	1.567	1.542	1.549 ± 0.012
	30	1.540	1.524	1.549	1.539	1.538 ± 0.010^*^
	300	1.510	1.508	1.483	1.500	1.500 ± 0.011^**^
**Benzo[a]pyrene (B[a]P)**	0	1.560	1.575	1.522	1.547	1.551 ± 0.021
	0.1	1.505	1.592	1.500	1.541	1.535 ± 0.039
	1	1.582	1.547	1.510	1.538	1.544 ± 0.027
	10	1.481	1.502	1.494	1.504	1.495 ± 0.010^**^
	100	1.205	1.464	1.470	1.429	1.392 ± 0.117^*^
**Fluorene-9-bisphenol (BHPF)**	0	1.541	1.509	1.512	1.572	1.534 ± 0.027
	0.005	1.550	1.481	1.515	1.571	1.529 ± 0.037
	0.05	1.521	1.479	1.473	1.599	1.518 ± 0.054
	0.5	1.500	1.452	1.465	1.555	1.493 ± 0.043
	5	1.401	1.429	1.391	1.462	1.421 ± 0.029^**^

Total values are mean ± SEM

Significantly different from control; *p* < 0.05*, *p* < 0.005*

### Time response

The most effective concentration confirmed for each PAH in the dose–response experiment was used to incubate the prepared cells for 24, 48, and 72 h (5% CO_2_, humidified atmosphere at 37 °C). The OD value for each incubation time was determined using the XTT assay.

### XTT assay

An XTT solution was prepared by dissolving 1 mL of activation reagent (sterile solution containing N-methyl dibenzopyrazine methyl sulfate) with 5 mL of XTT reagent at 37 °C immediately before use. Then, 50 µL of the solution was added to each well, incubated at 37 °C in a CO_2_ incubator for 3 h, and the plate was slowly shaken manually until the solution turned orange. The absorbance of the wells containing cells and blank background control was measured at 450–500 nm using a microtiter plate reader. The absorbance of the cell-containing wells and control wells was also measured at 630–690 nm to assess non-specific readings. We determined the average value from the triplicate readings and subtracted the average value for the blank wells as well as the average value of the non-specific readings. When performing the XTT assay, the following parameters were used:

- Specific absorbance filter: 475 nm
- Non-Specific absorbance filter: 660 nm

The specific absorbance of the sample was calculated using the following formula:

Specific absorbance = A_475nm_ (Test) − A_475nm_ (Blank) − A_660nm_ (Test).

### Statistical analysis

Each experimental group was conducted with the minimum number (*n* = 3) required for statistical analysis. The results were analyzed using Mann–Whitney test and Kruskal–Wallis test; *p* < 0.05 was considered statistically significant.

## Results

### Dose response

OD was measured at 450–650 nm. The absorbance before exposure to anthracene was 1.530 ± 0.029, but as the concentration increased, the OD value decreased. For anthracene at 0.5 µg/mL concentration, the OD was 1.470 ± 0.046, which was significantly lower than that before treatment (*p* < 0.05). When the cells were treated with 5 and 50 µg/mL anthracene, the OD values were also significantly lower than before the treatment (1.530 ± 0.029 vs. 1.420 ± 0.051, *p* < 0.005; 1.530 ± 0.029 vs. 1.366 ± 0.067, *p* < 0.005, respectively).

In HTR-8/SVneo cells treated with increasing benzo[k]fluoranthene concentrations (3 µg/mL), the absorbance decreased with an increase in concentration. When the concentration reached 30 µg/mL, there was a significant difference as compared to the effect before treatment (1.555 ± 0.010 vs. 1.538 ± 0.010, *p* < 0.05, respectively). After treatment with 300 µg/mL benzo[k]fluoranthene, the absorbance was 1.500 ± 0.011, which was significantly lower than that of the control (*p* < 0.005).

**Fig. 1:**
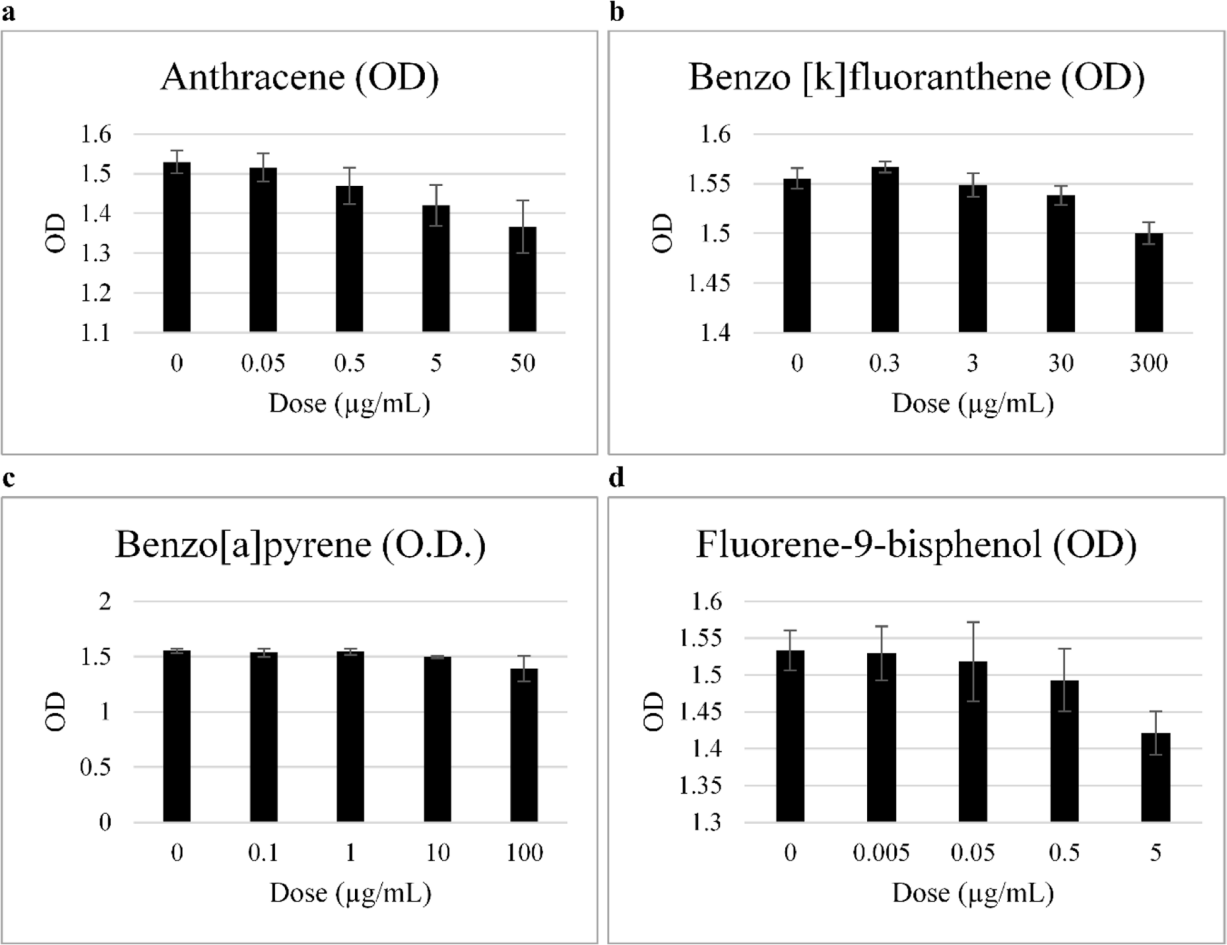
Dose response measured by the XTT assay (OD at 450–650 nm). HTR-8/SVneo cells were treated with anthracene, benzo[k]fluoranthene, benzo[a]pyrene, and 4,4-(9-fluorenylidene)diphenol for the cell viability analysis using XTT assay. The absorbance prior to anthracene exposure was 1.530 ± 0.029, which decreased with an increase in anthracene concentration. In HTR-8/SVneo cells treated with increasing concentrations of benzo[k]fluoranthene, the absorbance decreased with an increase in the concentration. Treatment with 300 µg/mL benzo[k]fluoranthene resulted in an OD of 1.500 ± 0.011, which was lower than that of the control. Following treatment with benzo[a]pyrene at concentrations of 10 µg/mL and 100 µg/mL, the absorbance was lower than that of the control. Treatment with 4,4-(9-fluorenylidene)diphenol, which is a substitute for BPA, showed a decrease in absorbance at a concentration of 5 µg/mL

With benzo[a]pyrene concentration of 10 µg/mL and 100 µg/mL, the absorbance was significantly lower than that of the control (1.551 ± 0.021 vs. 1.495 ± 0.010, *p* < 0.005; 1.551 ± 0.021 vs. 1.392 ± 0.117, *p* < 0.05, respectively). Treatment with 4,4-(9-fluorenylidene)diphenol, which is a substitute for BPA, showed a significant difference in absorbance when treated with 5 µg/mL (1.534 ± 0.027 vs. 1.421 ± 0.029, *p* < 0.005) (Table 1 and Fig. 1).

### Time response

**Fig. 2:**
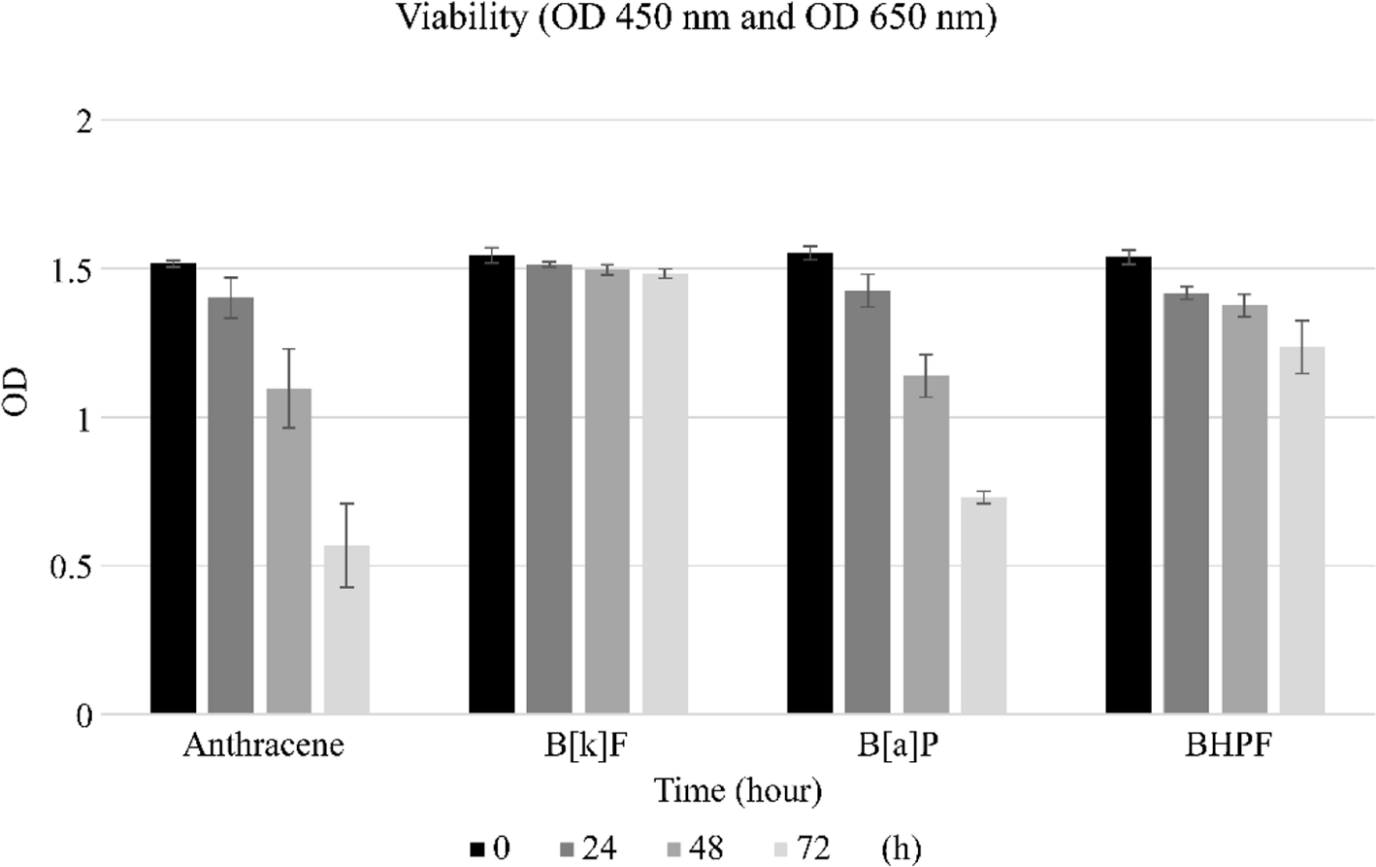
Time course in the XTT assay (OD at 450–650 nm). HTR-8/SVneo cells were treated with anthracene, benzo[k]fluoranthene, benzo[a]pyrene, and 4,4-(9-fluorenylidene)diphenol at 24, 48, and 72 h for the time course analysis using XTT assay. Treatment of the cells with anthracene (50 µg/mL) showed an initial absorbance of 1.517 ± 0.011, which showed a decreasing tendency with increasing time, decreasing to 1.402 ± 0.068 at 24 h, 1.097 ± 0.132 at 48 h, and 0.568 ± 0.141 at 72 h. The OD values obtained following 300 µg/mL benzo[k]fluoranthene treatment for 24, 48, and 72 h were also lower than the initial value. The absorbance following treatment with benzo [a]pyrene at a concentration of 100 μg/mL showed a decrease after 24 h. Similar results were observed when the cells were treated with 5 µg/mL of 4,4-(9-fluorenylidene)diphenol. Compared with the initial OD value, the absorbance was lower than that of the control at 24 h, 48 h, and 72 h

The change in absorbance over time was confirmed by treatment with the endocrine disruptor at the dose verified in the dose–response experiment (Table 2 and Fig. 2). The initial absorbance obtained following the treatment of HTR-8/SVneo cells with anthracene (50 µg/mL) was 1.517 ± 0.011, which showed a tendency to decrease to 1.402 ± 0.068 (*p* < 0.05) at 24 h, 1.097 ± 0.132 (*p* < 0.05) at 48 h, and 0.568 ± 0.141 (*p* < 0.005) at 72 h. The absorbance following treatment with 300 µg/mL of benzo[k]fluoranthene was also significantly lower than the initial value at 24, 48, and 72 h (1.545 ± 0.025 vs. 1.513 ± 0.010, p < 0.05; 1.545 ± 0.025 vs. 1.495 ± 0.017, *p* < 0.05; 1.545 ± 0.025 vs. 1.483 ± 0.015, p < 0.005). The absorbance for the treatment with benzo[a]pyrene at a concentration of 100 μg/mL, as confirmed by the XTT assay, showed a significant difference after 24 h (1.553 ± 0.023 vs. 1.426 ± 0.056, *p* < 0.005; 1.553 ± 0.023 vs. 1.139 ± 0.072, *p* < 0.005; 1.553 ± 0.023 vs. 0.730 ± 0.020, *p* < 0.005). Similar results were also observed when the cells were treated with 5 µg/mL of 4,4-(9-fluorenylidene)diphenol. When compared with the initial OD value, the absorbance was statistically significantly lower than that of the control at 24 h, 48 h, and 72 h (1.539 ± 0.025 vs. 1.419 ± 0.022, *p* < 0.005; 1.539 ± 0.025 vs. 1.376 ± 0.039, *p* < 0.005; 1.539 ± 0.025 vs. 1.236 ± 0.089, *p* < 0.005).

**Table 2:** Absorbance of HTR8/SVneo according to time course

Materials	Time course (h)	OD (Individuals)				OD (Total, *n* = 4)
**Control (DMSO only)**	0	1.537	1.590	1.515	1.588	1.558 ± 0.035
	24	1.72	1.784	1.707	1.812	1.756 ± 0.046
	48	1.995	2.013	1.92	2.146	2.019 ± 0.087
	72	2.008	2.104	1.971	2.231	2.079 ± 0.107
**Anthracene** **(50 µg/mL)**	0	1.520	1.511	1.532	1.506	1.517 ± 0.011
	24	1.373	1.451	1.470	1.312	1.402 ± 0.068^*^
	48	1.042	1.285	1.115	0.947	1.097 ± 0.132^**^
	72	0.495	0.702	0.388	0.685	0.568 ± 0.141^**^
**Benzo[k]fluoranthene (B[k]F)** **(300 µg/mL)**	0	1.562	1.519	1.563	1.524	1.545 ± 0.025
	24	1.522	1.500	1.521	1.509	1.513 ± 0.010^*^
	48	1.509	1.471	1.510	1.490	1.495 ± 0.017^*^
	72	1.478	1.469	1.507	1.477	1.483 ± 0.015^**^
**Benzo[a]pyrene (B[a]P)** **(100 µg/mL)**	0	1.582	1.560	1.549	1.522	1.553 ± 0.023
	24	1.450	1.441	1.475	1.338	1.426 ± 0.056^**^
	48	1.115	1.230	1.046	1.165	1.139 ± 0.072^**^
	72	0.701	0.726	0.749	0.745	0.730 ± 0.020^**^
**Fluorene-9-bisphenol (BHPF)** **(5 µg/mL)**	0	1.502	1.550	1.565	1.540	1.539 ± 0.025
	24	1.411	1.453	1.400	1.410	1.419 ± 0.022^**^
	48	1.380	1.414	1.317	1.392	1.376 ± 0.039^**^
	72	1.225	1.375	1.186	1.159	1.236 ± 0.089^**^

All values are mean ± SEM. Significantly different from control; *p* < 0.05*, *p* < 0.005**

## Discussion

In this study, we evaluated changes in the placental cell line treated with PAHs to determine the effects of PAHs on cell viability and necrosis. We found that the absorbance measured using the XTT assay decreased with an increase in the concentrations of PAHs and time in HTR-8/SVneo cells treated with anthracene, benzo[k]fluoranthene, benzo[a]pyrene, and 4,4′-(9-fluorenylidene)diphenol. This result indicates a positive correlation between the above PAHs and cell necrosis according to the dose and time in the placental cell line.

The placenta plays an important role in nutrient transport and fetal growth during pregnancy. The formation of a spiral artery from the placenta provides an uninterrupted supply of nutrient-rich maternal blood to the fetus by the end of the first trimester [11]. Human placental growth hormone, human chorionic gonadotropin, progesterone, estradiol, and placenta lactogen are secreted from the human placenta; all play a crucial role in pregnancy and fetal development [12]. Several studies have reported that exposure to PAHs in pregnant women may be associated with many changes in fetal and pregnancy outcomes, including pregnancy loss, changes in the onset of labor, such as preterm delivery, and abnormal fetal growth. In addition, it has been reported that the placenta may play a mediating role in these endocrine disruptors during pregnancy [13–17]. Our results showed that the placenta exposed to PAHs may be adversely affected, leading to complications during pregnancy.

In particular, the loss of cell viability in the placenta is associated with premature labor. We provide experimental evidence to prove that PAHs induce preterm labor by confirming that PAHs evoke necrosis of placental cells. In a study investigating the correlation between the PAH concentration in the placenta and preterm labor in women exposed to high PAH exposure, it was found that the concentration and DNA products of PAHs affect preterm labor. They reported that the concentrations of benzo[a]pyrene, benzo[b]fluorene, and dibenzo[a,h]anthracene were higher in the placenta of pregnant women with preterm delivery than in those with full-term delivery [18].

There are some limitations to our study. The HTR-8/SVneo cell line used in our study is considered a mixed cell line of trophoblast and stromal cells. The HTR-8/SV neo cell line has been known as the primary extravillous trophoblast in previous studies. Although recently considered and tested as a placenta cell line, there are still many studies that consider it as a trophoblast cell and lead to results. Therefore, we applied it to our study as it is. Nevertheless, additional experiments are needed to determine which cells are actually affected.

Despite such limitation, our study has strengths. PAHs are stabilizers and plasticizers found in many consumer products, including toys and hygiene products. They are also used in blood bags, medical devices, adhesives, solvents, and pesticides [19, 20]. BPA and phthalate metabolites are detected in urinalysis in more than 90% of pregnant women in the United States because of their high PAH exposure [21]. However, the effects of endocrine disruptors such as PAHs on the placenta have not been sufficiently studied despite pregnant women being exposed to them almost daily. In addition, most studies have focused only on BPA. Exposure to PAHs during pregnancy is an essential issue in teratogenicity and preterm birth, and adverse perinatal outcome continues to increase. Our study is meaningful in that it provides the basis for research on the correlation between PAHs and preterm birth by providing evidence that PAH exposure causes placental cell necrosis. Furthermore, our study showed an important finding that BPA substitutes are also unsafe PAHs.

## Conclusions

Our study showed that anthracene, benzo[k]fluoranthene, benzo[a]pyrene, and 4,4′-(9-fluorenylidene)diphenol, a BPA substitute that has been insufficiently studied, affect cell proliferation and differentiation in a placental cell line. This means that these PAHs cause cell necrosis in the placenta, leading to adverse perinatal outcomes, such as preterm birth. These results can be used as basic data for future research related to preterm birth and PAHs.

## Data Availability

The datasets used and analyzed during the current study are available from the corresponding author on reasonable request.

## Abbreviations

PAH: Polycyclic aromatic hydrocarbons
BPA: Bisphenol A
OD: Optical density

## References

[1] Sanderson JT (2006). The steroid hormone biosynthesis pathway as a target for endocrine-disrupting chemicals. Toxicol Sci.

[2] Sweeney T (2002). Is exposure to endocrine disrupting compounds during fetal/post-natal development affecting the reproductive potential of farm animals?. Domest Anim Endocrinol.

[3] Patel S, Zhou C, Rattan S, Flaws JA (2015). Effects of Endocrine-Disrupting Chemicals on the Ovary. Biol Reprod.

[4] Woodruff TK, Walker CL (2008). Fetal and early postnatal environmental exposures and reproductive health effects in the female. Fertil Steril.

[5] Boström CE, Gerde P, Hanberg A, Jernström B, Johansson C, Kyrklund T (2002). Cancer risk assessment, indicators, and guidelines for polycyclic aromatic hydrocarbons in the ambient air. Environ Health Perspect.

[6] Miller KP, Borgeest C, Greenfeld C, Tomic D, Flaws JA (2004). In utero effects of chemicals on reproductive tissues in females. Toxicol Appl Pharmacol.

[7] Perera FP, Rauh V, Tsai WY, Kinney P, Camann D, Barr D (2003). Effects of transplacental exposure to environmental pollutants on birth outcomes in a multiethnic population. Environ Health Perspect.

[8] Kim A, Park M, Yoon TK, Lee WS, Ko JJ, Lee K (2011). Maternal exposure to benzo[b]fluoranthene disturbs reproductive performance in male offspring mice. Toxicol Lett.

[9] Le Vee M, Kolasa E, Jouan E, Collet N, Fardel O (2014). Differentiation of human placental BeWo cells by the environmental contaminant benzo(a)pyrene. Chem Biol Interact.

[10] Drwal E, Rak A, Grochowalski A, Milewicz T, Gregoraszczuk EL (2017). Cell-specific and dose-dependent effects of PAHs on proliferation, cell cycle, and apoptosis protein expression and hormone secretion by placental cell lines. Toxicol Lett.

[11] Rattan S, Zhou C, Chiang C, Mahalingam S, Brehm E, Flaws JA (2017). Exposure to endocrine disruptors during adulthood: consequences for female fertility. J Endocrinol.

[12] Kaufmann P, Black S, Huppertz B (2003). Endovascular trophoblast invasion: implications for the pathogenesis of intrauterine growth retardation and preeclampsia. Biol Reprod.

[13] Woodruff TJ, Zota AR, Schwartz JM (2011). Environmental chemicals in pregnant women in the United States: NHANES 2003–2004. Environ Health Perspect.

[14] Veiga-Lopez A, Kannan K, Liao C, Ye W, Domino SE, Padmanabhan V (2015). Gender-Specific Effects on Gestational Length and Birth Weight by Early Pregnancy BPA Exposure. J Clin Endocrinol Metab.

[15] Adibi JJ, Hauser R, Williams PL, Whyatt RM, Calafat AM, Nelson H (2009). Maternal urinary metabolites of Di-(2-Ethylhexyl) phthalate in relation to the timing of labor in a US multicenter pregnancy cohort study. Am J Epidemiol.

[16] Ferguson KK, McElrath TF, Ko YA, Mukherjee B, Meeker JD (2014). Variability in urinary phthalate metabolite levels across pregnancy and sensitive windows of exposure for the risk of preterm birth. Environ Int.

[17] Felsenfeld G (2014). A brief history of epigenetics. Cold Spring Harb Perspect Biol.

[18] Suter MA, Aagaard KM, Coarfa C, Robertson M, Zhou G, Jackson BP (2019). Association between elevated placental polycyclic aromatic hydrocarbons (PAHs) and PAH-DNA adducts from Superfund sites in Harris County, and increased risk of preterm birth (PTB). Biochem Biophys Res Commun.

[19] Mervish N, McGovern KJ, Teitelbaum SL, Pinney SM, Windham GC, Biro FM (2014). Dietary predictors of urinary environmental biomarkers in young girls, BCERP, 2004–7. Environ Res.

[20] Newbern D, Freemark M (2011). Placental hormones and the control of maternal metabolism and fetal growth. Curr Opin Endocrinol Diabetes Obes.

[21] Heudorf U, Mersch-Sundermann V, Angerer J (2007). Phthalates: toxicology and exposure. Int J Hyg Environ Health.

